# Social life of free-living amoebae in aquatic environment— comprehensive insights into interactions of free-living amoebae with neighboring microorganisms

**DOI:** 10.3389/fmicb.2024.1382075

**Published:** 2024-06-19

**Authors:** Shi Fan, Yun Shen, Li Qian

**Affiliations:** Department of Civil and Environmental Engineering, School of Engineering and Applied Science, The George Washington University, Washington, DC, United States

**Keywords:** free-living amoebae, FLA, bacteria, viruses, microorganisms, eukaryotes

## Abstract

Free-living amoebae (FLA) are prevalent in nature and man-made environments, and they can survive in harsh conditions by forming cysts. Studies have discovered that some FLA species are able to show pathogenicity to human health, leading to severe infections of central nervous systems, eyes, etc. with an extremely low rate of recovery. Therefore, it is imperative to establish a surveillance framework for FLA in environmental habitats. While many studies investigated the risks of independent FLA, interactions between FLA and surrounding microorganisms determined microbial communities in ecosystems and further largely influenced public health. Here we systematically discussed the interactions between FLA and different types of microorganisms and corresponding influences on behaviors and health risks of FLA in the environment. Specifically, bacteria, viruses, and eukaryotes can interact with FLA and cause either enhanced or inhibited effects on FLA infectivity, along with microorganism community changes. Therefore, considering the co-existence of FLA and other microorganisms in the environment is of great importance for reducing environmental health risks.

## 1 Introduction

Free-living amoebae (FLA) are a type of eukaryotic organisms that can be widely present in different environments. Unlike prokaryotic unicellular organisms such as bacteria and archaea, FLA have more complicated cell structures. Compared with bacteria or viruses, this type of microorganisms has nucleus packing their DNA and abundant organelles. These features allow them to differ from bacteria or viruses in interaction behavior and mechanism with other microorganisms in the environment. Compared to parasitic amoebae, FLA can survive and reproduce independently without hosts. They inhabit a diverse range of habitats, such as water, soil, air, and even extreme environments (Rivera et al., 1987; Visvesvara and Stehr-Green, 1990). The two main life stages of FLA are the trophozoite (active, feeding stage) and the cyst (dormant, resistant stage). Since FLA reproduce under favorable conditions and differentiate into dormant forms under unfavorable conditions, FLA can persist in different harsh conditions without perishing (De Jonckheere, 1991; Khan et al., 2015). However, this may not always happen when they are treated in some extreme cases (e.g., boiling). In addition, FLA are also prevalent in man-made environments such as swimming pools, sewage, cooling water, eyewash solutions, contact lenses, dialysis units, and dental treatment units (Mergeryan, 1991; Water, 2010). To date, FLA include four genera known to contain pathogenic species: *Acanthamoeba, Balamuthia, Naegleria*, and *Sappinia* (Schuster and Visvesvara, 2004). The T4 genotype of *Acanthamoeba* is implicated in various severe human pathogenic diseases, including granulomatous amebic encephalitis (GAE), a fatal infection of the central nervous system (Otero-Ruiz et al., 2022). *Acanthamoeba* keratitis (AK), primarily affecting long-term contact lens wearers, has been reported in over 3,000 patients worldwide so far (Stehr-Green et al., 1989; Juarez et al., 2018). Moreover, *Balamuthia mandrillaris* and *Sappinia pedata* can also cause amebic encephalitis, referred to as *Balamuthia* amebic encephalitis (BAE) and *Sappinia* amebic encephalitis (SAE), respectively. Among the ~100 reported cases of BAE, there have been only three known survivors (Qvarnstrom et al., 2013; Luca, 2022). Likewise, *Naegleria fowleri* can cause primary amebic meningoencephalitis (PAM), a disease with a notably high fatality rate, typically contracted through exposure to contaminated water sources (Capewell et al., 2015).

Given the considerable pathogenic threats posed by FLA to human health, it is important to comprehensively understand how FLA behave in real environments and to mitigate the associated public health risks. Achieving this goal requires a deeper understanding of FLA within intricate ecosystems. While extensive research has explored amoeba growth, reproduction, mutation, and pathogenicity with human infection, the relationship between FLA and microbial communities has received limited attention.

Studies have highlighted that interactions between FLA and other microorganisms can also facilitate the transmission of diseases caused by other types of microorganisms. FLA play an important role in the disposal of organisms by acting as a “Trojan horse” that serves as a vector for the colonization of new habitats or hosts (Vaerewijck et al., 2014). FLA typically use bacteria and other microorganisms as food sources, but some prey species can escape predation and survive within the host or even proliferate within its cytoplasm or nucleus, which is called amoeba-resistant microorganisms (ARM) (Rayamajhee et al., 2021). Some eukaryotes even reversely cleave FLA as a host to obtain their nutrients (Steenbergen et al., 2003). Hence, a comprehensive review of the interplay between FLA and environmental microorganisms is imperative for future studies in microbial risk management.

Therefore, here we aim to (1) systematically review the various common types of microbial groups that can interact with FLA; (2) understand the different patterns and mechanisms of interactions between these microorganisms and FLA, so as to understand the neighboring impact to FLA in complex ecological environments. This review will provide crucial information for public health forecasting.

## 2 Interactions between FLA and bacteria

### 2.1 The impact of FLA on bacterial growth and community change

FLA influence the survival, growth, and community of bacteria in water through predation. Studies have shown that FLA can discern and selectively target certain bacteria as food sources while avoiding pathogenic ones (Bornier et al., 2021). This preferential hunting behavior significantly shapes the co-existing bacterial community. *Acanthamoeba castellanii* displays a marked preference for consuming bacteria from the *Betaproteobacteria* class and *Firmicutes* phylum in soil systems, leading to a notable decrease in these dominant bacterial taxa and a concurrent increase in other grazing-tolerant bacteria (Rosenberg et al., 2009). Moreover, this influence on bacterial communities can also pose a threat to public health. In engineered water systems, it has been shown that FLA have a preference for consuming bacteria from the environment, with an order of *Escherichia coli* strains and then *Legionella pneumophila*. However, *L. pneumophila* are not indeed “consumed,” since they can use FLA as a Trojan horse and replicate within them (Shaheen and Ashbolt, 2021). This will also lead to a change in population ratios for microorganisms in water. Prey bacteria for FLA will decrease first with an increase of FLA, which will later facilitate the growth and replication of other pathogenic bacteria like *L. pneumophila* (Shaheen and Ashbolt, 2021). Therefore, FLA have a significant impact on controlling bacterial populations and altering community composition.

Some bacteria show resistance to FLA predation and their growth can be even supported by the existence of FLA. These bacteria are known as amoeba-resistant bacteria (ARB). Typically, ARB can survive and be protected within FLA, and the detection of ARB correlates with FLA abundance in environments (Delafont et al., 2016; Malinowski et al., 2022). For example, *Legionella* spp. have been reported to strongly associate with FLA in potable water distribution systems from 10 hospitals in southwest France, with this relationship being partly temperature-dependent (Lasheras et al., 2006). A previous study also revealed a statistically significant co-occurrence of *Legionella* spp. and FLA, with all *Legionella*-positive samples tested positive for FLA (Nisar et al., 2022). Gene expression may also be altered for *L. pneumophila* during the interaction with FLA (Buse et al., 2015). The study showed that interactions between three strains of *L. pneumophila* and FLA hosts were promoted after gray water exposure, with an inhibition of amoebal encystment and an alteration of bacterial gene expression. In addition, biofilms can greatly impact the interactions between *Legionella* and FLA. The densities of *Legionella* spp. positively correlated with *Vermamoeba vermiformis* densities (*r* = 0.83, *p* < 0.028) in biofilms within garden hoses (Thomas et al., 2014). It was reported that FLA, including *Willaertia magna* and *Acanthamoeba polyphaga*, showed long-term interactions with *L. pneumophila* in natural biofilms (Shaheen et al., 2019). A nutrient recycling within the system of FLA and culturable *Legionella* can lead to a balance in concentrations of FLA and *Legionella* at room temperature, especially after a 85-week's co-culturing. And this can allow the detection of *Legionella* even after a quite long period.

*Mycobacterium* is another significant group of ARB. The growth of *Mycobacterium avium* was markedly enhanced in the presence of *A. castellanii*. Notably, these bacteria also exhibited increased virulence when infecting beige mice after growth within amoebae (Cirillo et al., 1997). A strong association between FLA and nontuberculous mycobacteria has also been observed in drinking water distribution systems (Delafont et al., 2014). Specifically, *Acanthamoeba, Vermamoeba, Echinamoeba*, and *Protacanthamoeba* have been shown to carry and support the replication of mycobacteria, including *Mycobacterium llatzerense* and *Mycobacterium chelonae*. The mechanism for *M. avium* surviving intracellularly in amoeba has also been proposed. Upon *Acanthamoeba* infection, *M. avium* rapidly enters individual vacuoles that subsequently merge into a single large vacuole. By inhibiting lysosomal fusion akin to macrophages, *M. avium* can survive and replicate within amoeba cells. *M. avium* replicates at temperatures as low as 24°C and that its growth and interaction with FLA can enhance virulence (Rubenina et al., 2017). Some *Pseudomonas* can also be supported by FLA. It was shown that viable but non-culturable (VBNC) *Pseudomonas aeruginosa* could be even resuscitated to an active form within 2 h when associated with *A. polyphaga* (Dey et al., 2019).

Finally, FLA can also facilitate the resistance of bacterial pathogens toward disinfectants in drinking water. It was revealed that the resistance of *M. avium* to water disinfectant monochloramine was largely enhanced when bacteria were co-cultured with *Acanthamoeba* (Berry et al., 2010). Moreover, the effectiveness of chlorine and heat treatments to *Legionella* spp. strains were observed to be reduced when *Legionella* was associated with *Acanthamoeba* (Cervero-Aragó et al., 2015). However, the supporting effect is not the only way FLA influence co-existing bacterial species. It was reported that an inhibition effect was surprisingly observed for *Willaertia magna* c2c that the growth of one strain of *L. pneumophila* was inhibited, while other similar strains did not show this effect (Dey et al., 2009). Therefore, since many pathogenic bacteria can be supported when associated with FLA, FLA can thus be considered as a shelter for bacteria, and can further increase the health risks caused by associated bacterial pathogens.

**Figure 1:**
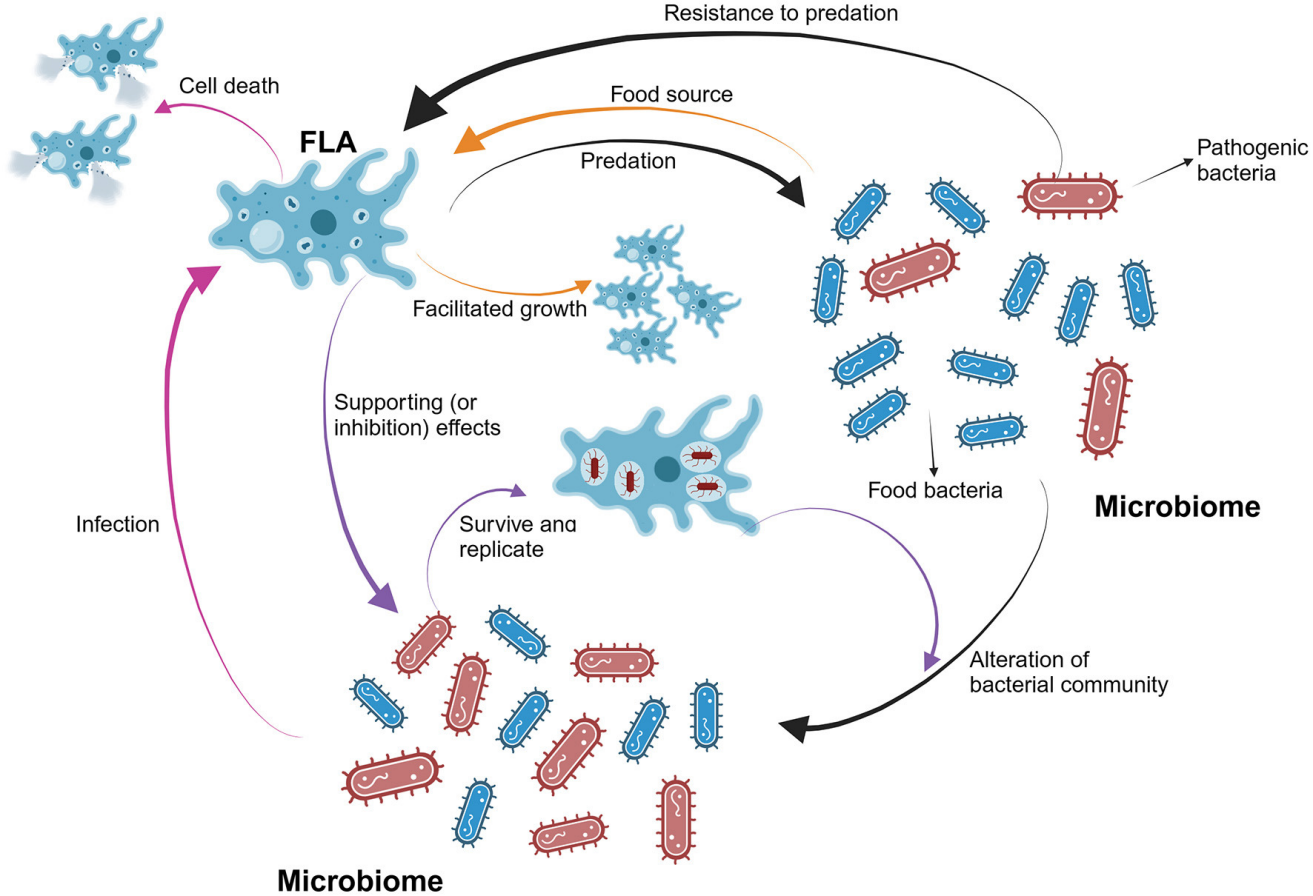
The overall interactions between FLA and bacteria in water systems. FLA can selectively consume food bacteria and thereby alter the populations of different bacteria species in the bacterial community. The abundance of FLA will increase first when sufficient food bacteria are available. Also, pathogenic bacteria can resist the predation and infect FLA. Serving as a harbor for pathogenic bacteria, FLA will facilitate the growth and replications of them. At this point, the abundance of FLA will decrease due to the induced death caused by the pathogenic bacteria.

Overall, FLA can influence the occurrence and abundance of bacteria by acting as a predator and a harbor (Figure 1). It is particularly important to note that the presence of FLA in engineered water systems can substantially increase the occurrence of opportunistic bacterial pathogens, including *Legionella* and *Mycobacterium*, posing risks to public health. Thus, establishing more robust monitoring and detection methods for FLA in water distribution systems is crucial.

### 2.2 The impact of bacteria on FLA survival

The growth of FLA could be supported by co-culturing with bacteria. It has been shown that the external bacteria enhance *Acanthamoeba* growth and binding to contact lenses, as well as the survival of amoebae in biocidal lens care solutions (Bottone et al., 1992). However, this is not always the case. It is reported that at low densities, gram-negative bacteria in PBS supported *A. castellanii* growth. However, at amoebae to bacteria ratios exceeding 1:10,000, gram-negative bacteria, including *E. coli*, became inhibitory to the amoebae (Wang and Ahearn, 1997).

Certain bacterial species, especially the pathogenic ones, can alter the life cycles of FLA. *L. pneumophila* was reported to induce the death of FLA after replication within them (Dey et al., 2009). Different *L. pneumophila* strains have been shown to inhibit the growth of amoebae of *A. castellanii, Hartmannella vermiformis*, and *W. magna*, although susceptibility varies among different amoebae. The bacteria of *Burkholderia cepacian* was observed to slightly reduce the viability and cyst formation of *A. polyphaga* in co-cultures (Anacarso et al., 2010). The bacteria of *Pseudomonas* strains demonstrated a clear type of endocytosis inside amoebae, with *Pseudomonas fluorescens* causing lysis of the host, whereas *P. aeruginosa* did not (Anacarso et al., 2010). FLA were found to show dependence on their endosymbionts. It was reported that symbiotic bacteria could provide essential cellular functions to amoebae, compensating for genetic defects in housekeeping genes caused by the bacteria themselves (Choi et al., 1997). In summary, some pathogenic bacteria cause significant damage to FLA cells during their intracellular replication, leading to a decrease in the viability and abundance of FLA communities.

## 3 Interactions between FLA and viruses

### 3.1 FLA as hosts for giant viruses

**Figure 2:**
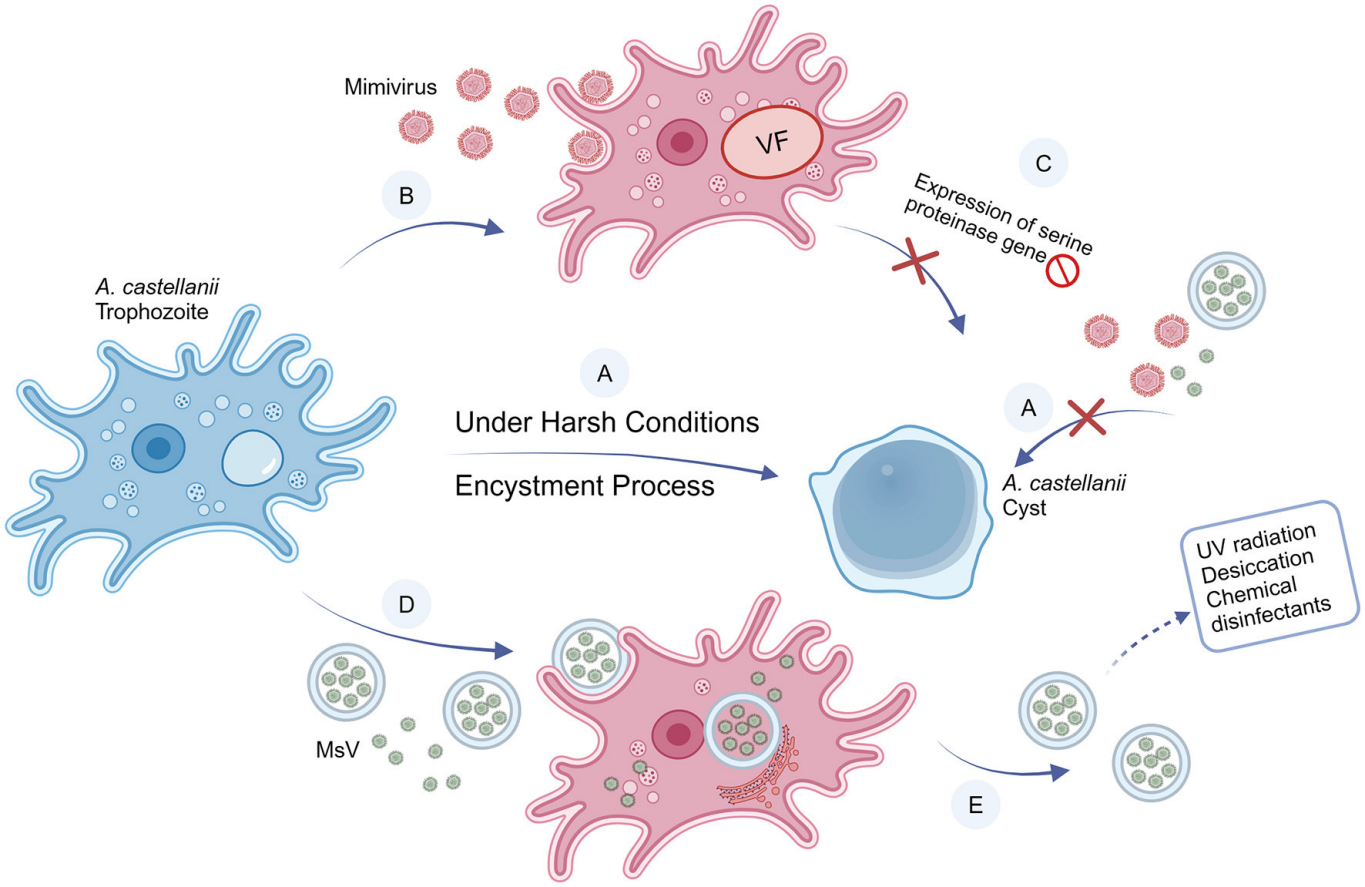
Interactions between *A. castellanii* and mimivirus and MsV. **(A)** Under harsh conditions (i.e., extreme temperatures, hunger, unfavorable pH, etc), *A. castellanii* develops a coping strategy by differentiating from trophozoites (vegetative form) into cysts (resistant form), a process called encystment. Once triggered encystment, the giant viruses are unable to infect cysts. **(B)** Mimiviruses can infect and replicate in *A. castellanii* trophozoites. **(C)** When *A. castellanii* is infected, the mimivirus blocks the expression of the serine proteinase gene, preventing the encystment process, thereby the cyst cannot form. **(D)** Compared with a single MsV particle, the size of vesicles containing MsV particles is large enough to trigger phagocytosis of *A. castellanii*. Particles can be released into the trophozoite as individual particles or vesicles, thereby initiating infection. **(E)** Compared with free particles, vesicles can increase infectivity and improve resistance to the external environment.

The discovery of giant viruses has highlighted a new dimension of complexity in the study of amoebae (Balczun and Scheid, 2017). The structure of giant viruses is far more complex than ordinary viruses. Most giant viruses interact with host FLA through infecting their trophozoites. Initiating the amoeboid phagocytosis process is necessary for these viruses to start their replication cycle and facilitate the formation of their progeny (Raoult and Boyer, 2010). This enhanced comprehension of giant viruses' dynamics with amoebae paves the way for an in-depth examination of particular instances, exemplified by mimiviruses, which redefine conventional notions of viral morphology and activity while underscoring their complex symbiosis with amebic hosts. Here, we illustrated the interactions between typical giant viruses and their FLA hosts in Figure 2.

Mimiviruses, distinguishable by light microscope due to their coccobacillus-like structure, constitute a unique group of substantial viruses. The *Acanthamoeba polyphaga mimivirus* (APMV), colloquially referred to as mimivirus, was first isolated from the cytoplasm of amoebae (Suzan-Monti et al., 2007), marking its first recognition as a viral entity. Subsequent research identified mimiviruses as having a circular, double-stranded DNA genome of more than 1 million base pairs (Raoult et al., 2004). Beyond their large genome size, mimiviruses are unique for their departure from the typical viral reliance on host cell machinery for protein translation, showcasing greater complexity and autonomy. Simply, mimiviruses can replicate in amoebae rather than being digested. Unlike most other viruses, mimiviruses actively transcribe their genome while proliferating in its FLA hosts, suggesting an active utilization of their genetic information, instead of passive replication (Suzan-Monti et al., 2007; Colson et al., 2017).

Another giant virus, *Marseillevirus* (MsV), was also isolated from *Acanthamoeba*. MsV shares a very similar icosahedral structure with mimivirus (Boyer et al., 2009), but is much smaller (<500 nm). A free MsV would not enter FLA through phagocytosis, because the phagocytosis is triggered when FLA recognize particles >500 nm (Weisman and Korn, 1967). Instead, MsV can enter FLA through enormous vesicles derived from the endoplasmic reticulum (ER). It was found that these vesicles consisted of various numbers of membranes. These vesicles may contain at least 10 virus particles. When the vesicles are single-layered, they can merge with the FLA cell membrane, and release the viruses directly into the cytoplasm. In multi-layered vesicles, a similar fusion process occurs, but the inner membrane remains intact, containing a cluster of viral particles. This novel entry mechanism of MsV via giant vesicles represents an innovative mode of large DNA virus invasion into host cells (Arantes et al., 2016). Giant vesicles could confer resistance to MsV against environmental factors, boost the replicative success of MsV within its natural hosts, and facilitate MsV transmission in the environment. This phagocytosis process provides an avenue for small-sized viruses, which will otherwise go unnoticed by FLA, to invade host cells.

While mimiviruses and MsV grow and thrive in specific FLA, there are more complex giant viruses infecting a wide range of FLA. Tupanvirus, known as one of the most complex giant viruses, was found to infect *Acanthamoeba castellanii, Acanthamoeba polyphaga, Acanthamoeba griffini*, and *Vermamoeba vermiformis* (Abrahão et al., 2018). Tupanvirus can manipulate the host cells in a peculiar way which is known as the Tupanvirus-induced cytopathic effect (CPE) to continually provide a site for their progeny to grow and proliferate. Tupanvirus encodes a mannose-specific lectin (MBP) that activates cellular and viral mannose receptor genes, enhancing FLA cell attachment (Barre et al., 1997). Tupanvirus-infected FLA cells mix with uninfected cells to form large bundles of host cells, boosting the likelihood that viral progeny finds additional host cells (Oliveira et al., 2019).

### 3.2 Emerging interactions with viruses

In addition to giant viruses, several other viruses were also found to interact with FLA. *Reovirus*, for example, can be internalized into and enriched in the nucleus of trophozoites within FLA (Silverstein et al., 1976). Accumulation of *Reovirus* particles in the nucleus after phagocytosis does not impede the normal growth of trophozoites. Instead, *Reovirus* can persist as aggregates. This peculiar symbiotic relationship allows *Reovirus* to successfully find a harbor suitable for their own survival, thus easily avoiding the threat of external disinfection (Folkins et al., 2020). Similarly, human adenovirus type 5 (HAdV5), Norovirus, and others can also be internalized by FLA to avoid physical and chemical disinfection processes (Hsueh and Gibson, 2015; Verani et al., 2016).

While FLA were shown to harbor non-enveloped viruses, the interactions between enveloped viruses and FLA were rarely studied. To explore the possible interactions between FLA and enveloped severe acute respiratory syndrome coronavirus 2 (SARS-CoV-2), surrogate experiments using the enveloped bacteriophage Phi6 were conducted to investigate the vectorial capacity of *V. vermiformis*. Phi6's induction of apoptosis and impact on amoeboid mitochondria, facilitating trophoblast persistence, might suggest a mechanism for SARS-CoV-2's interaction with FLA (Dey et al., 2022).

Overall, understanding how giant viruses interact with FLA hosts, has revealed the viral complexity and diversity. The intricate mechanisms of infection and replication challenge the conventional paradigms of viral-host dynamics. The observation of cytopathic effects signifies an advanced level of host-virus interaction. Furthermore, understanding the dynamics of these viral entities can shed light on the mechanisms of pathogen persistence and transmission, offering insights into the risks they may pose to human health, especially in the context of waterborne and zoonotic diseases.

## 4 Interactions between FLA, fungi, and protozoans

FLA can also interact with numerous eukaryotes. One such group, referred to as amoebophagous fungi, can function either as predators or parasites of their prey, the FLA (Corsaro et al., 2018; Scheid, 2018). In particular, dimorphic fungi, which existed in the form of mold or yeast, were found to kill FLA and use them as a source of nourishment through cell lysis. For example, *Cryptococcus neoformans*, a human pathogenic fungus, exhibits sustained growth when co-cultivated with amoebae over an extended period of time. In detail, *Dictyostelium discoideum*, one amoeba host, engulfed *C. neoformans* cells, and the presence of budding fungal cells inside *D. discoideum* suggested that *C. neoformans* cells replicated intracellularly (Steenbergen et al., 2003). Nevertheless, those fungi can not stay extensively within FLA. Instead, they can break down the FLA cells and grow extracellularly (Steenbergen et al., 2003). This process involves killing and attacking the host cells from inside and then extracting the required nutrients from the remains (Steenbergen et al., 2004). Additionally, it is important to point out that FLA do not only rely on phagocytosis to engage with dimorphic fungi. *Blastomyces dermatitidis* primarily utilizes proteins on the membrane surface to adhere to the FLA cell membrane surface, resulting in cytotoxic effects on the FLA and ultimately causing its demise (Steenbergen et al., 2004). In addition, it was found that the fungi increased their virulence against the great wax borer which was used as an infection model for the pathogenic dimorphic fungi, through the transmission of *A. castellanii* (Thomaz et al., 2013). Therefore, the complex and predatory interactions between FLA and dimorphic fungi significantly affect ecosystems and human health, potentially enhancing fungal virulence and influencing infection spread.

In addition to fungi, FLA can interact with other protozoan parasites. Specifically, certain FLA, such as *Acanthamoeba* and *Naegleria*, may serve as potential hosts for oocysts of *Toxoplasma gondii*. The *T. gondii* oocysts taken up by *A. castellanii* were found to maintain their capacity to cause an infection in mice (Winiecka-Krusnell et al., 2009). The same study also suggested that *T. gondii* oocysts might block lysosomal binding thus allowing themselves to escape digestion.

In conclusion, the predatory behavior of dimorphic fungi toward FLA and the ability of FLA to potentially harbor and interact with protozoan parasites highlight possible pathways for disease transmission and the enhancement of pathogen virulence. The multifaceted interactions between FLA and a range of eukaryotic organisms underscore a critical microbial ecology with direct implications for human health and environmental stability.

## 5 Discussion

FLA play a significant role in microbial ecosystems within natural and engineered water environments, where their interactions with other microorganisms profoundly influence community dynamics and health outcomes. This review provides an overview of documented interactions between FLA and various microorganisms across taxonomic divisions. For bacteria and some fungi, FLA exhibit predatory behavior toward them, which serve as a food source, while parasitism represents another mode of interaction with certain bacteria and fungi. Current research has demonstrated FLA serve dual roles for pathogenic bacteria by providing a protective environment and facilitating their competitive adaptation (Rubenina et al., 2017), yet significant knowledge gaps remain. Despite studies have reported how the infection of FLA by bacteria like *L. pneumophila* or *M. avium* occurs, the mechanism of surviving and evolving within FLA for many other bacteria still largely remains unclear. In particular, it remains uncertain whether FLA can provide a unique niche to select certain mutations or induce genetic drift and transfer in bacterial populations. The future study on the evolution and transmission of bacterial pathogens within FLA is needed. While exogenous bacterial interactions with FLA have been extensively studied, exploring endogenous bacterial environments within FLA presents an intriguing avenue for investigation. These environments create specific micro-ecosystems where various ARB evolve within FLA cells. The evolution may be associated with horizontal gene transfers within FLA, and it is also worth investigating whether ARB can acquire antibiotic resistance genes (ARG) from other bacteria. Various methods can be utilized for exploring the bacteria within FLA. In particular, metagenomics may help in determining the ARG they contain, as well as the evolution of wide-ranged pathogenic bacteria, due to its broad applicability for unknown microbes. Additionally, FLA's mobility may facilitate the transport of pathogenic bacteria in the environment. Despite the relevance to public health risks, its influence on bacterial fate and transport remains understudied. Therefore, future research efforts could prioritize the study of ARB evolution and transmission within FLA.

It is crucial to conduct further studies on how human viruses coexist with FLA in the environment, but there are gaps in our current understanding of the interactions between viruses and FLA. Focusing on key factors such as environmental conditions to streamline variables, and ensuring consistent experimental setups to minimize heterogeneity, such research will deepen our knowledge of these interactions and help develop targeted strategies for mitigating virus-related risks. Meanwhile, the mechanism of gaining resistance for virus harboring in FLA is largely neglected. For instance, giant viruses have the ability to reproduce within FLA and simultaneously modify the host's internal environment to support their propagation. This necessitates further investigation into molecular mechanisms, such as whether giant viruses utilize a ribosome-independent translation system. Such a system would allow them to synthesize proteins more efficiently without competing for the host's ribosomes, potentially explaining their enhanced replication within FLA. Regarding the fungal aspect, there is currently very little research on the targeted application of amoebophagous fungi as biocontrol tools in environments where abundant pathogenic FLA exists (Scheid, 2018). However, this type of research is crucial, especially for the application of such fungi with no impact on human health.

Prior research has established a solid base of data and valuable insights into the interactions between FLA and other microorganisms. Future efforts must address the research gaps resulting from the limited diversity and mechanism study of microorganisms examined.

## Author contributions

SF: Writing – review & editing, Writing – original draft. YS: Writing – review & editing, Writing – original draft. LQ: Writing – review & editing, Writing – original draft.
